# Performance and suitability analysis of rooftop solar PV in Oman: A case study of university branches

**DOI:** 10.1016/j.heliyon.2025.e42578

**Published:** 2025-02-11

**Authors:** Sharmila deve Venkatachalam, Almuhannad Al Nadabi, Abdul Aziz Al Shukaili, Ahmed Said Al Hinai, Ahmed Salim Al Shuaili, Ibrahim said Al Shukaili

**Affiliations:** College of Engineering and Technology, University of Technology and Applied Sciences, Ibri Branch, Ibri, Sultanate of Oman

**Keywords:** Rooftop solar PV, Performance analysis, Levelized cost of electricity, System advisor model, Policy frameworks, Sultanate of Oman

## Abstract

One of the goals of Oman vision 2040 is to attain a 30 % of renewable energy mix, mainly from solar and wind energy projects for electricity generation by 2030, in alignment with the net zero emissions commitment by 2050. The adoption of residential rooftop solar PV installations supports achieving this target. This paper aims to assess the feasibility and performance of the rooftop solar PV projects at various locations in Oman and to suggest the strategies for promoting rooftop solar PV projects in Oman. In the Middle East countries like Oman, dust from sandstorms and temperature are significantly affects the performance of PV systems and is an important derating factor to consider when evaluating their performance as discussed in this paper. This paper starts by qualitatively assess the suitable regions in Oman for solar PV projects based on temperature levels, dust accumulation, humidity and population density and then proceeds to find the best locations in the selected region. A rooftop solar PV system is designed, analysed its performance, Levelized Cost of Electricity (LCOE) and environmental benefit were calculated for smart bus stop load located in the selected region's university of technology and applied sciences (UTAS) campus using analytical method. The results obtained through analytical method were compared with System Advisor Model (SAM) software. Key findings from the study: The northern part regions of Oman were identified as the most suitable region to install the solar PV systems. In the northern part regions, Ibri is the best location for rooftop solar PV projects compared to other locations. However, at other locations, the AC energy generation from 2.2kWp solar PV systems ranges from 4243.44 kWh to 4841.34 kWh annually, shows that these locations are also competitively suitable for solar PV projects. The LCOE and carbon emission cost of all UTAS branch locations ranges from 8.5 ¢/kWh to 9.7¢/kWh and 128.16 $ per year to 146.74 $ per year accordingly. After finding the most suitable locations and analysis, attractive rooftop policies in solar PV of successful countries are discussed, based on that few strong strategies are suggested to develop rooftop solar PV projects in Oman. The findings from this study will provide the useful guidelines for Oman's energy sector and policymakers to implement and promote roof top solar PV projects in Oman.

## Graphical abstract

**Figure:**
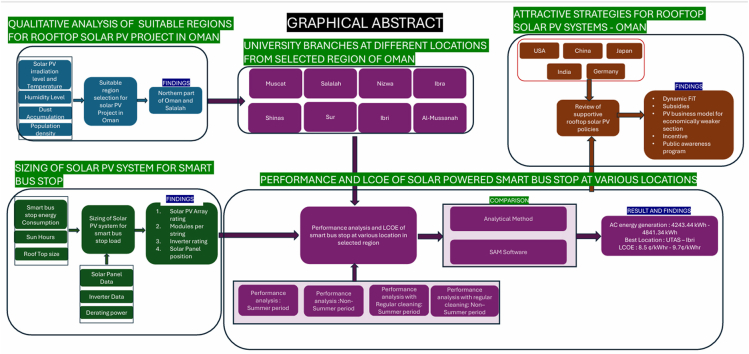

## 1 Introduction

The Climate change is the biggest threat to our planet, like a war between humanity and nature. However, in this battle, humanity's survival requires working alongside nature than confronting it. Hence, to address climate change key steps have been taken in the Paris agreement and COP 28 summit. As per the Paris Agreement, a target was set to limit global warming to 1.5 °C. This target has led to set plans to triple renewable energy capacity and double energy efficiency by 2030 at 2023 COP 28 summit [1]. As a result, many countries have established the 2030 goals, including reducing emissions and taking steps to mitigate the climate change impacts.

A key aspect of these plans involves mixing renewable energy sources into the energy sector, eventually reducing the fossil fuel usage. Consequently, many countries are launching many renewable energy projects, introducing new renewable energy schemes, incentives, and policies to attract public interest, raising renewable energy research funding and creating public awareness about climate change. This global shift shows the responsibility of every individual role in protecting our environment and ensuring a sustainable future. Driven by concerns about climate change and global warming, the global PV cumulative capacity grew to 1.6 TW by 2023. China and the United States lead the global PV market, with 760 and 265 GW of installed capacity, respectively. The installed capacity in Africa amounted to approximately 221 GW [2].

As per the Paris Agreement, the Sultanate of Oman is committed to reduce greenhouse gas (GHG) emissions and incorporating renewable energy mix in their energy sector. Based on this commitment, the country has planned a target to achieve net zero emissions by 2050 [3]. To achieve this target, Oman energy sector has initiated various projects to incorporate renewable energy sources. The country also aims for 11 % renewable energy in its energy mix by 2025 and 30 % by 2030 [3] to achieve the Oman vision 2040 renewable energy goal and the 2050 goal in timely manner. In line, with the Paris Agreement commitments Oman aims to reduce its absolute greenhouse gas emission by 7 % by 2030 as outlined in its climate action strategy [4]. The National Strategy for an orderly transition to net zero states that emission reduction rate of 54 % in 2040 and 92 % in 2050 from 2021 values.

The Sultanate of Oman is fortunate to have renewable energy sources such, as solar power, wind power, geothermal energy and ocean energy. In global market, among the renewable energy sources, solar PV is preferred due to its advancement of its technology and decreasing costs over the year compared to other renewable energy and hybrid energy sources. Additionally, its size flexibility, easy of installation, low maintenance, and longevity contribute to the significant growth in the global market compared to other conventional and renewable energy sources. Furthermore, its robustness and reliability in energy production through various hybrid sources, such as PV-grid, PV-wind, PV -diesel, PV – concentrated solar power, PV-wind-diesel and PV-wind-battery systems, made it widespread deployment globally, which results global cumulative capacity of solar PV grew to 1.6 TW by 2023 [[5], [6], [7], [8], [9], [10], [11], [12]]. Electricity can be generated from solar PV technology and solar thermal system. Although, the Levelized Cost of Energy for solar thermal system is lower than solar PV technology in arid regions like the Kingdom of Saudi Arabia, solar PV is preferred due to its size of flexibility, which allows widespread adoption in the residential and utility-scale applications [13]. Hence, when it comes to renewable energy sources Omans's energy sector primarily focuses on photovoltaic (PV) generation than other renewable energy source. Also, Oman has implemented policies for rooftop PV systems such as Sahim rooftop solar PV initiative [14] and several solar projects have been initiated since 2020 that are expected to be operational, before 2030 [2].

Many researchers have analysed the utilization of solar PV in various applications, such as street lighting, maritime energy solutions, desalination plant, grid stability, and industrial energy optimization, all of which contributes the sustainability, economic growth and environmental protection [[15], [16], [17], [18], [19], [20]]. Consequently, numerous studies have explored the potential of solar PV in different locations, the feasibility of rooftop solar PV, public awareness of the solar PV transition, policies to promote solar PV and the overall scope of solar energy in Oman.

Oman region is classified as a desert with high dust accumulation, Al siyabi et al. conducted an experimental analysis on the effect of soiling on a 2MWp of car park PV plant at Muscat, Oman and their result shows that 5.6 % monthly electricity generation reduced by 7.5 % of soiling percentage and 10.8 % generation reduced by 12.5 % of soiling-percentage [21]. Dust accumulation on solar PV was tested in six cities of Northern Oman and it was found that Liwa, Sohar and Muscat exhibit the higher percentage of dust accumulation due to industrial activity and more vehicles makes the air pollution. In contrast, Al-Khabourh, Suwaiq, and Shinas, which are far away from the industries and limited vehicles experienced the limited dust accumulation. Hussein A. Kazem and Miqdam T. Chaichan recommended that sodium solution is the best option to clean the solar PV in industrial cities, while water washing is sufficient for the other cities [22].

The performance of Solar PV cell material in desert regions is different from that in other regions. The best suitable solar PV system for Oman and the best solar PV site among 25 locations in Oman were identified using HOMER software. The research found that the best type of PV is the Ingeteam 1164kVA with generic PV. The best suitable solar PV sites are Marmul followed by Fahud, Sohar, and Qairoon Hairiti, due to their relatively low Cost of Energy, high clearness index and high level of solar radiation [23].

A total of 130 modules were located in three different region such as Moderate climate, Hot and Humid climate, Hot and Dry Climate places and conducted field study to analyze the degradation rate in these regions. The findings indicated that higher degradation rates and Encapsulant discoloration were observed in hot and humid, hot and dry areas. This study recorded that 1.96 % per year of degradation rate and 93 % of old age panels observed with Encapsulant discoloration. Based on the research findings Honnurvali, Mohamed Shaik recommended that organic PV cell material exhibit higher temperature sensitivity than traditional PV cell material. Additionally, he recommended to avoid Encapsulant discoloration and delamination in PV modules can be avoided by using strong adhesive strength material between the glass and Ethylene Vinyl Acetate (EVA) [24]. A grid tied 1.4kWp solar desert type PV system was installed at sultan Qaboos University, Muscat. It was monitored for a year and recorded the results for analysis. The result revealed that the monthly average daily capacity factor reached 17 % which is higher compared to similar systems installed in other locations world-wide. This study also investigated the impact of dust on the desert type PV, which showed that the percentage of annual energy reduction was only 10 % [25].

Zero Energy building (ZEB) is one of the modern concepts for energy saving strategy, solar PV is one of the main components used in ZEB. In Oman, as a demo Zero energy building is constructed and solar PV performance on ZEB was analysed in some research papers [26,27]. A 20 kW solar PV is installed on the rooftop of ZEB building located at Sultan Qaboos university and analysed the building energy performance and energy balance. The result showed that the building was less than 3 % from achieving its net zero building status [28]. AL Badi investigated the performance and dust impact on the Eco house rooftop solar PV. The results illustrated that, since the rooftop solar PV is placed in a low dust accumulation area, the percentage of energy reduction is minimal. Additionally, the author compared its performance results with other international researchers. The findings revealed that the average daily capacity factor reached 15 %, which is either higher or similar to other systems installed in various locations worldwide.

A rooftop PV-grid independent system is feasible in Oman by considering reduction of energy demand per household, the introduction of support policies and a reduction in battery costs [29,30]. A 1 MW grid connected solar PV in Adam city, Oman was assessed. The assessment results proved that the selected location is promising for solar PV investment as it has good annual energy yield and capacity factor [31].

Valuable insights for Oman's energy sector policymakers to forecast potential growth, identify effective renewable energy policy instruments and evaluate public interest in the solar energy transition are important for shaping the renewable energy mix in energy sector, as assessed in Ref. [32]. The results revealed that 95 % of residents and commercial units are willing to use solar PV in the future. The authors identified the main barriers are high installation cost, high maintenance cost and lack of awareness.

Accurate solar irradiance estimation is an important factor to improve solar field efficiency and energy yield. Yasser F. Nassar et al. developed a model and estimated solar irradiance on solar fields [[33], [34], [35], [36]]. In this paper, key design parameters are considered to design a smart bus stop solar PV.

The major electricity consumption in Oman is residential sector [37]. Rooftop solar PV supports greatly during daytime peak loads and summer period loads [38]. Raising public interest and awareness about rooftop solar PV can be effectively achieved by installing systems at commercial places and academic institutions. Since students represent the future of the country, equipping them with hands-on experience of solar PV technology at their universities or schools not only educate them but also contributes spread awareness among the general population. This approach can facilitate the seamless adoption of rooftop PV systems in residential areas.

Previous research papers related to solar projects in Oman has primarily focused on the rooftop solar PV case studies at single location, analysing their performance. However, Oman's diverse geographical and climate at each location plays crucial role in the efficient performance of solar PV systems. This paper addresses this gap. Previous research studies evaluated the performance of solar PV on annual basis. However, Oman experiences varying sun hours, residential load and temperature during summer and non-summer period. In this paper, performance of solar PV is assessed separately for these two periods instead of annually. Previous studies analysed the impact of dust on solar panel, however not focused on sandstorm. Oman frequently experiences sandstorms. This paper examines the dust impact due to sandstorm along with the impact of cleaning, on solar PV performance during summer and non-summer period at various locations.

This paper provides a step-by-step approach, starts from selecting the suitable locations for solar PV projects to proposing successful strategies for the adoption of rooftop solar energy project in the residential sector of Oman. As Oman is a desert country, the study starts by assessing the factors that affect solar PV performance in desert regions, followed by qualitatively identifying the suitable regions in Oman for rooftop solar PV project. After identifying the suitable regions, a detailed case study was conducted to design and assess the rooftop solar PV systems performance in the selected regions. This case study first focuses on the design, performance analysis of a rooftop solar PV system for a smart bus stop located at UTAS-Ibri branch. The performance analysis and LCOE assessment were conducted and compared for the virtual solar-powered smart bus stop across various UTAS branches located in Northern region and Dhofar region of Oman. Finally, the results are discussed and compared with SAM results, and successful solar polices from other countries are reviewed. Based on these, a few strong strategies are suggested to promote the solar PV projects in Oman.

## 2 Qualitative assessment of suitable regions for solar PV projects across Oman

Oman is blessed with abundant solar and wind energy resources, which strengthens the confidence to establish the solar PV systems throughout the Sultanate of Oman. It experiences the average 8 h of sunshine per day during winter and up to15 h per day during summer, with an average radiation per day approximately 5 kWh/m^2^ [39]. The country has an excellent solar intensity, with over 342 sunny days per year [40]. Solar PV generation depends not only on solar irradiation but also on the relative humidity of a location. Higher relative humidity reduces solar PV output. In Oman, coastal regions experience higher relative humidity during summer compared to the dry inland regions, which reduces the PV output. Frequent sand and dust storms in Oman, reduces the intensity of solar radiation, resulting in decreased solar power generation [[41], [42], [43]]. Oman geographical features, such as mountains, arid deserts, limited water resources, and challenging terrain contributes to a sparse population and lower energy demand. Therefore, the following main factors are considered to find the suitable region for solar PV projects in Oman: solar irradiation level, temperature coefficient, humidity levels, dust impact and population density. In this section, the factors affecting solar PV projects are discussed and a qualitatively assessment of suitable regions across Oman is presented.

### 2.1 Solar PV irradiation level and temperature coefficient

**Fig. 1:**
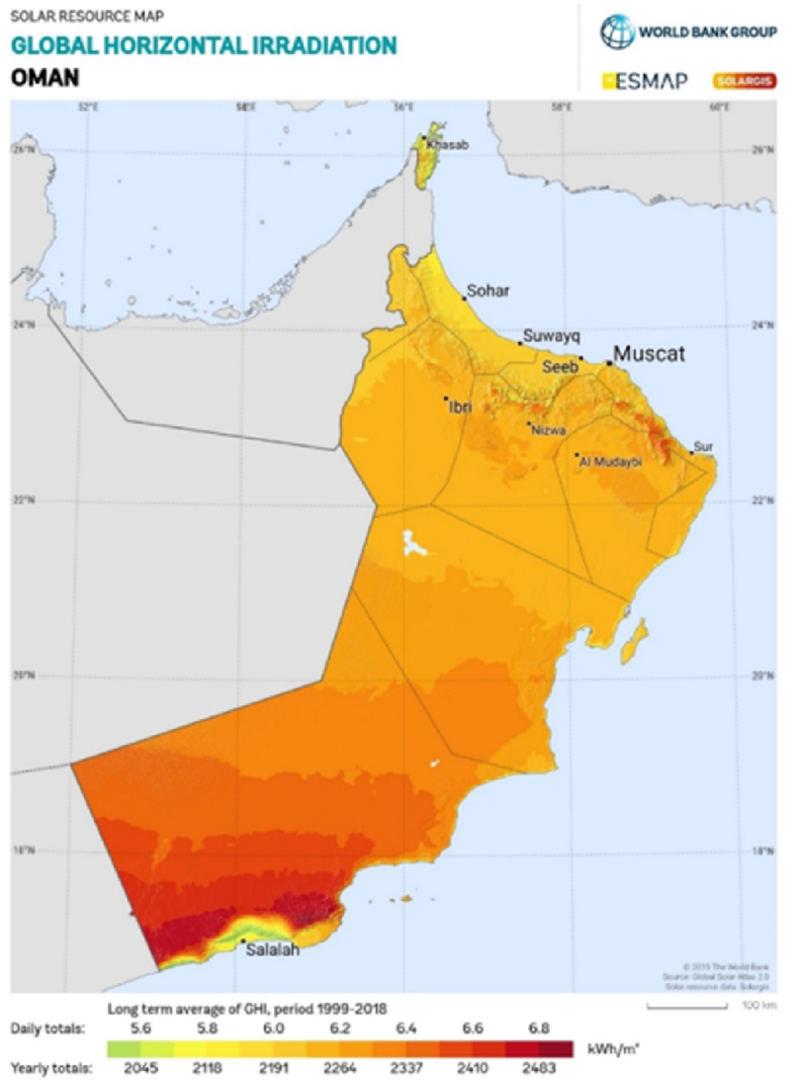
Global Horizontal Irradiation (GHI) of Oman [44].

Source: Solar resource map © 2021 Solargis.

Fig. 1 depicts the global horizontal irradiation (GHI) period of Oman, showing that this country has high irradiation level. This spatial graph is sourced from SolarGIS [44]. Normally, the solar irradiation level required to generate considerable electrical power from solar PV panel ranges between 100 and 200 W/m^2^ [45]. The daily irradiation level across the country ranges from minimum 4.7 kWh/m^2^/day to a maximum 7.470 kWh/m^2^/day [24]. These values are highly favourable to produce electrical power from solar panels. In hot regions, this rooftop solar PV system reduces direct solar radiation on the roof and minimizing heat transfer into the building [46].

Additionally, Fig. 1 highlights that the minimum and maximum solar irradiation levels are experienced in the Dhofar region. Salalah receives the minimum GHI compared to other locations. Though it receives minimum GHI, that itself enough to produce significant electrical power from solar panels. The main areas like Muscat, Nizwa, Ibri and Sohar receive more than 2000 kWh/m^2^ annually.

Solar PV output depends on the GHI. Practically, a simplified model [13] is used to calculate solar PV DC output (Pdc),

(1) $$P_{dc}=P_{rated}*\frac{GHI}{G_{STC}}*\left[1+\beta \left(T_{cell}-{T\;}_{ref}\right)\right]$$

Where:

P_DC_ - DC power output of the PV panel in W.

P_rated_ - Rated power capacity of the PV panel at Standard Test Conditions (STC) in W.

GHI - Global Horizontal Irradiance at the location in W/m^2^

G_STC_ - Solar irradiance at STC, typically 1000 W/m^2^

β - Temperature coefficient of power for the PV module (per °C). This is typically a negative value, around −0.004 to −0.005 per °C for most silicon-based panels.

T_cell_ - Operating temperature of the PV cell in °C.

T_ref_ - Reference temperature at STC in °C.

Solar PV panel output degrades due to the operating temperature of PV cell. Therefore, Oman's high temperature cause greater degradation of solar PV output compared to European Countries [47].

Temperatures, across the country fluctuate due to a mix of terrains and oceanic effects. The highland regions in the north and south generally moderate temperatures, throughout the year [48]. In Oman the highest temperatures fall between 32 °C and 48 °C, both during daytime and nighttime are typically experienced in May and August. On the other hand, the lowest air temperatures ranging from 10 °C to 23 °C between December and February. Notably the inner and central deserts of Oman exhibit peak daytime temperatures around 50 °C during summer.

Temperature significantly influences the PV panel performance and its overall efficiency [49], Although Oman has excellent solar irradiation, due to its high temperature levels, the northern part and Dhofar are more suitable for solar PV installations than the inner and central deserts of Oman.

### 2.2 Humidity level

The existence of moisture in the atmospheric air scatters the sunlight, disrupts the path of solar irradiation falling on the solar surface, resulting in reduced solar PV output performance [50]. This situation can impact the efficiency of panels situated in regions, with consistently high humidity levels like coastal area in Oman.

**Fig. 2:**
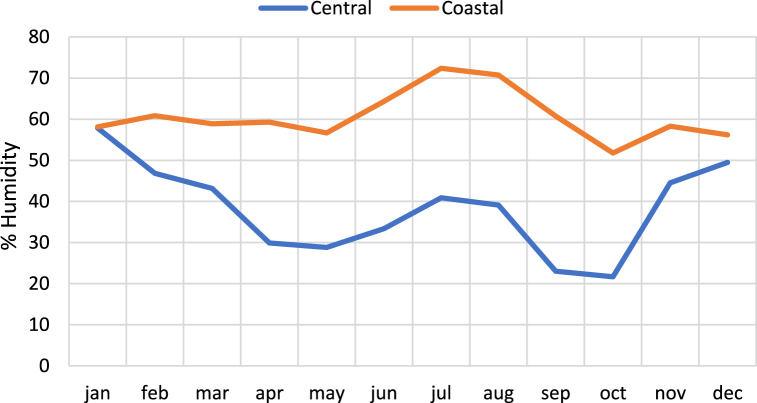
Average Humidity of Central areas and Coastal Areas of Oman [51]. Source: NCSI, Oman.

A study conducted by Ref. [39] observed that coastal regions experience high humidity in winter, while the Arabian Sea coasts have high humidity levels, exceeding 85 % during summer in Oman. Fig. 2 shows the average humidity levels, in Central Oman and Coastal areas during the year of 2020. Data taken from National centre for statics and Information (NCSI), Oman [51]. It highlights that the coastal region experiences more humidity compared to the central region. This study suggests that interior regions are more favourable than coastal areas, for implementing PV projects.

### 2.3 Dust accumulation

Dust accumulation and soiling of solar panel affects the solar PV performance significantly [52]. In Oman, there are many factors to cause dust accumulation, such as emissions from power plant chimneys, smelters from the industry, movement of vehicle and sandstorms. Middle east countries often experience heavy sandstorms, which lead to more dust particles settling on solar panels, which severely affects their performance. Sandstorms are a more dangerous factor than others in affecting solar PV performance in Middle East countries. They have a large influence in reducing the solar radiation to reach the ground due to air turbidity and cause heavy dust deposition on solar panels [43].

Omans land area covers a total of 309,500 km featuring topographic elements including valleys and desert that make up 82 % of the land mountain ranges occupying 15 % and coastal regions comprising 3 %. The vast desert terrain significantly influences the country's climate and environment leading to sand and dust storms. The expansive desert areas pose a challenge when it comes to PV installation due, to the exposure to sand dust. Fine sand particles are easily stirred by winds. When the surface is heavily encrusted stronger winds are required for sand movement to occur [53].

Oman has different types of sand across its regions shown in Table 1. The Rub'al Khali and Wahiba sands are the primary types, with their fine and loose texture that make them easily swept by the wind. Moreover, there are other sand types are coarser and denser, in nature and necessitate stronger winds to be shifted around. The average wind speed, in Oman ranges from 10 to 20 km/h throughout the year. It can escalate during summer and winter with occurrences of Shamal winds. These winds carry an amount of dust and sand. During the summer season they often bring hotter weather conditions [54].

**Table 1:** Types of Sand and its characteristics in Oman.

Types of Sand	Characteristics	Areas covered	Impact of dust
Rub'al Khali (Empty Quarter) [56]	Fine, reddish sand.	Thumrait, Southern Oman	More
Wahiba Sands (Sharqiya Sands) [57]	Fine to medium-grained sand.	Bidiyah, sur, North east corner	More
Coastal Areas [58]	Coarse, white sand	Al-Batinah North and South, Muscat, Ash Sharqiya South, Al-Wusta, Dhofar, Musandam	Medium
Desert Plains and Gravel Deserts	Mixed coarse sand and gravel.	Ibra, Adam, Ibri, Buraimi	Medium
Mountain Regions	Coarse sand with rock fragments	Nizwa, Rustaq	Less
Alluvial Fans and Wadis	Silty sand and fine gravel	Sohar, Buraimi, Nizwa, Rustaq	Medium

Thickness and volume of the dust on the solar panel decides the dust impact on the solar panel performance. Nasser et al. [43] defined the thickness (Td) and volume (Vd) of dust on the solar panel.

(2) $$Td=\frac{volume\;of\;dust\;collected}{area\;of\;soalr\;panel}$$

(3) $$Vd=\frac{mass\;of\;dust\;collected}{desnisty\;of\;dust}$$

From equations (2) and (3), fine and loose textured sands easily accumulate on the solar panel and forms thick dust layer on the solar panel and reducing its efficiency. Based on the sand type, the impact of dust is analysed using equations (2) and (3), described as a linguistic variable, and summarised in Table 1.

Majority of Oman's topography consists of Rub’ al Khali and Wahiba sands, those areas may not be suitable for installing solar panels due to strong sand deposition. Areas with coarse, gravel sand are considered more suitable for solar PV projects. In these areas, dust accumulation on solar panels due to industrial emissions and vehicle movement. Regular cleaning of solar panels with water can help remove normal dust deposition. If sand deposition more on the solar PV panels, cleaning can be done effectively by applying electrostatic force methodology [55].

### 2.4 Population density

**Fig. 3:**
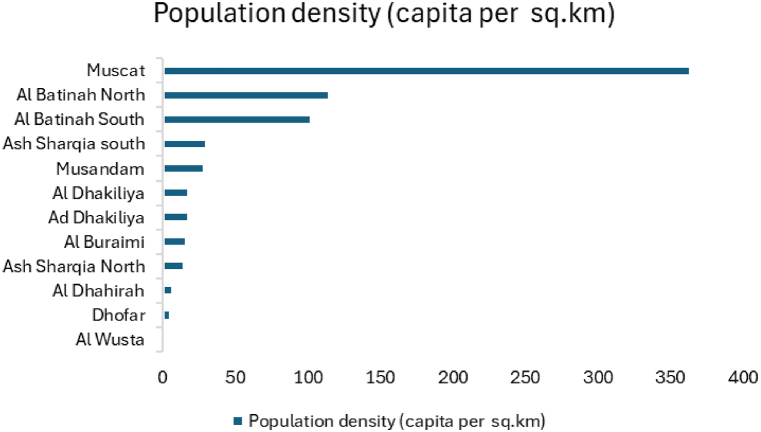
Population density of Oman [51]. Source: NCSI, Oman.

Population density is one of the factors used to decide the market of roof top solar PV. Densely populated areas require residential solar PV projects to meet the peak demand and ensure grid stability, while off-grid solar PV is suitable for remote areas. In 2024 Omans current population stands at 5.3 million with a population density of 17 peoples, per km^2^ [51]. The majority of the population is concentrated in Northern Oman while coastal areas have a moderate population density. The central and interior regions of Oman have very low population densities due to their predominantly desert landscape and harsh climate shown in Fig. 3. The highest population concentration is in the capital, Muscat. Most of the cities are in the northern part of Oman and Salalah in Dhofar region has the highest population density. The Northern part of Oman, coastal areas and Salalah are the most populated areas in Oman [59], which are suitable for solar PV projects. Off-grid solar PV projects are suitable for the central areas of Oman.

### 2.5 Discussion on solar PV project suitability in various regions across Oman

By considering factors such as solar irradiance, temperature, humidity levels, dust deposition and population density, the solar PV project suitability in various regions of Oman is analysed and summarised in Table 2. The most suitable locations for Solar PV projects are cities in the Northern part of the Oman and coastal areas due to their high solar irradiance and population density. These areas, with well-developed infrastructure, support the integration and maintenance of solar PV systems. Coastal areas have higher humidity than inland areas. The coarse, white sand and gravelly sand in these regions require strong winds to be deposit on solar PV panels. The humidity and dust due to sand, industry smelters and vehicle movement can be manageable with proper cleaning and the latest technology.

**Table 2:** Solar PV project suitability in various regions across Oman.

Region	Solar Irradiance	Humidity Level	Dust Level	Population Density	Suitability
Northern Oman	High	Moderate (higher near coast, lower inland)	Moderate to High	High	High
Southern Oman (Dhofar, Salalah)	Moderate to High (varies with season)	High during Khareef, moderate otherwise	Low to Moderate	Low to Moderate (highest in Salalah)	Moderate
Central Oman	High	Low	High	Very low	Low
Western Oman	Moderate to High	Moderate to High near coast, low inland	Low to Moderate	Low	Moderate
Coastal Areas	High	High	Moderate	High	High
Mountain Areas	Moderate to High (depending on altitude and location)	Low to Moderate	Low	Low	Low to Moderate

The Dhofar and Salalah regions experience high humidity during the Khareef season, while temperature and dust accumulation are moderate, making these regions moderately suitable for solar PV projects. Also, Salalah is one of the attractive tourist cities and the third most populated city in Oman, makes solar PV projects economically viable due to its active economic environment.

By considering solar irradiance and low humidity, mountain regions and central Oman are suitable for solar PV projects. But the low population density and high temperature makes solar PV projects less feasible and economically unviable. However, off-grid solar PV projects could be feasible in these areas to provide electricity for the lesser population.

## 3 Design and assess the rooftop solar PV system performance in the selected regions

**Fig. 4:**
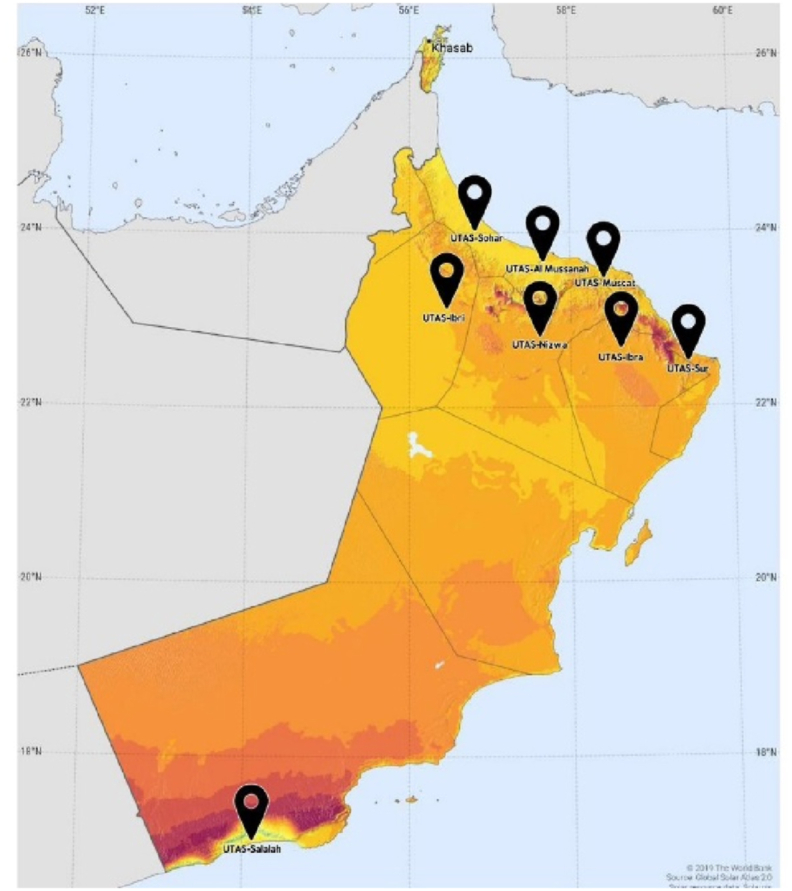
UTAS Branches Location in Oman.

Source: Solar resource map © 2021 Solargis.

Based on Table 2, Northern Oman regions and Salalah in Dhofar region are identified as the most suitable regions for solar PV projects. To assess the solar PV performance in each suitable region, eight UTAS branches were selected for case study due to their strategic locations. Table 3 and Fig. 4 presents the name and locations of the UTAS branches across Oman. A solar PV powered smart bus stop is designed for UTAS-Ibri branch, and their performance is analysed using analytical method.

**Table 3:** Solar PV for smart bus stop at various UTAS Branches across Oman.

UTAS Branch Name	Location, Region	Latitude	Longitude
UTAS – Muscat	Al Khoud, Muscat	23.5803	58.4328
UTAS – Salalah	Salalah, Dhofar	17.0473	54.1427
UTAS – Nizwa	Nizwa, Ad Dakhiliyah	22.8903	57.5560
UTAS – Ibra	Ibra, Ash Sharqiyah North	22.7764	58.4934
UTAS – Shinas	Shinas, Al Batinah North	24.7422	56.4292
UTAS – Sur	Sur, Ash Sharqiyah South	22.5667	59.4715
UTAS – Ibri	Ibri, Ad Dhahirah	23.2424	56.4196
UTAS - Al Mussanah	Al Mussanah, Al Batinah South	23.7432	57.5779

### 3.1 Energy demand of the smart bus stop

This bus stop is planned to be located within the university campus, Ibri and serves as a waiting area for female students. Its usage depends on the university's working hours typically starting with classes at 8 a.m. and ending at 4.00 p.m. During breaks between classes female students can sit inside the smart bus stop. The main electrical loads of this bus stop include a 1-ton smart AC unit, smart LED lights, a water dispenser and a LED TV. The AC unit and LED lights are equipped with motion sensors. When students occupy the bus stop, all these loads operate at capacity. However, if no students are inside, the AC automatically switches off while the LED lights continue to operate at low intensity.

The bus stop is usually occupied by students from 10 a.m. to 4 p.m. each day. The operating hours of AC and water dispenser are determined based on when students are available in the bus stop prevailing weather conditions especially during peak summer months and summer vacation holidays such as July and August. In peak summer periods like May and June, when temperatures soar high, the AC and water dispenser have longer operating hours to ensure comfort for the occupants. On the other hand, during vacation holidays in July and August when fewer students use the bus stop power consumption is minimized.

**Fig. 5:**
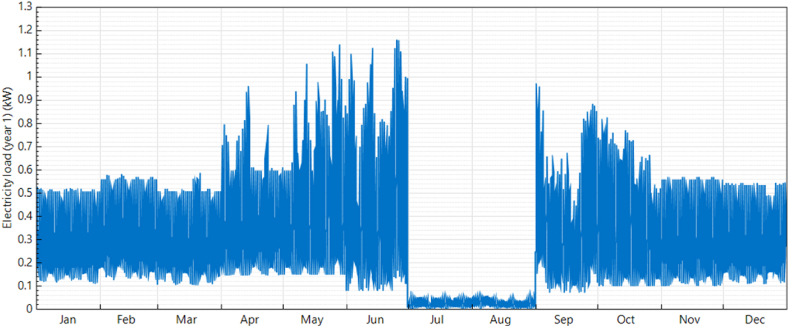
Electricity Daily load profile of smart bus stop.

The energy needs of the bus stop are categorized into four groups according to their power consumption as outlined in Table 4 and the one-year load profile is shown in Fig. 5. Table 4 displays the operating hours and power consumption for each month at this bus stop. During vacation periods, it is estimated that the bus stop uses 17.73 kWh of power per month. However, during peak summer months, power consumption significantly increases to approximately 324.22 kWh.

**Table 4:** Solar powered smart bus stop energy demand.

Load	Number in Use	Power Rating (W)	Winter (Nov–Mar)		Summer (Apr, Sep, Oct)		Peak Summer (May, Jun)		Vacation (Jul, Aug)	
			T∗ (H)	E∗∗ (W- H)	T∗ (H)	E∗∗ (W- H)	T∗ (H)	E∗∗ (W- H)	T∗ (H)	E∗∗ (W- H)
Smart AC	1	1000	7	7000	10	10000	13	13000	0	0
Smart LED light	10	18	13	2340	13	2340	13	2340	4.5	810
Water Dispenser	1	50	7	350	10	500	13	650	0	0
Smart TV	1	17	13	221	13	221	13	221	4.5	76.5
Daily Energy Consumption (kW- H)				9.911		13.061		16.211		8.86
Monthly Energy Consumption (kW- H)				198.22		261.22		324.22		17.73

T∗: Operating Duration, E∗∗: Energy consumed.

## 4 Design of solar PV system for smart bus stop load

### 4.1 Sizing of solar PV panel

**Fig. 6:**
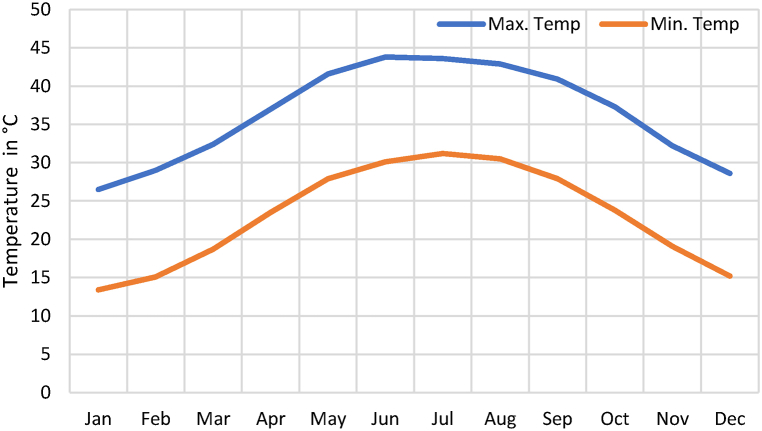
Average temperature, ibri. Source: Directorate general of Meteorology, Oman.

**Fig. 7:**
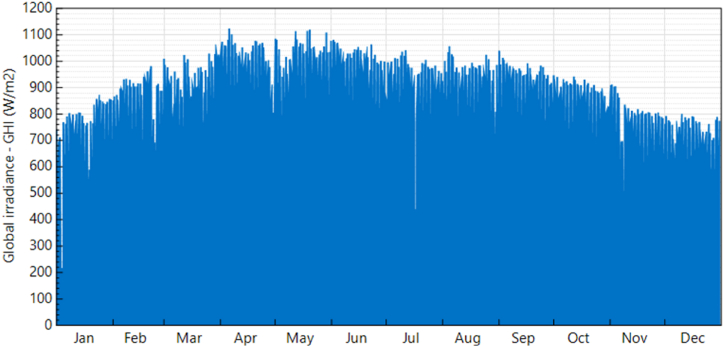
Global horizontal Irradiance (GHI), Ibri. Source: SAM weather files.

The daily power consumption of the smart bus stop depends on climatic conditions and university working hours. During peak summer, energy consumption of smart bus stop is more due to the continuous operation of heavy loads. While designing the solar PV system, peak summer energy consumption is considered for calculation. Ibri solar weather data and Global horizontal Irradiance (GHI), Ibri are shown in Fig. 6, Fig. 7 accordingly.

The system loss compensation factor accounts for all the losses in the solar PV system, from solar irradiance on the solar PV panel to the final AC output power to the load. Temperature losses, dust and dirt losses are significant due to high temperatures and frequent sandstorms in Oman [60]. These losses and their typical values are listed in Table 5. Shading loss is minimal due to the lack of cloud formation. Total system losses are approximately between 15 % and 25 % [[61], [62], [63]]. For this calculation, a 20 % system loss is considered.

**Table 5:** Types of losses and percentage ranges of losses in Solar PV system [[61], [62], [63]].

Type of Loss	Description	% range of loss
Reflection Losses	The glass on solar PV surface reflects the part of solar irradiance on it.	∼2–3%
Temperature Losses	Increased temperature reduces the open circuit voltage much more significant.	∼5–10 %
DC Cable Losses $P_{cable-loss}$	Due to resistance of the wires	∼1–3%
Inverter Losses	During conversion process of DC to AC	∼2–5%
AC Cable Losses	Due to resistance of the wires	∼1–2%
Shading Losses	Shading due to trees, buildings and clouds.	∼0–1%
Losses due to Dust and Dirt	Accumulation of dust due to sandstorm, Industries, and Vehicles	∼2–6%
Module Mismatch	Differences in the performance between individual solar panels within a string	∼1–3%

In solar PV calculations, size of the solar PV and the number of panels required depends on the daily demand requirement (E_daily_), loss compensation factor (PV_systemloss_), size of the module and sun hours per day (Hr_sun_) [15]. The demand requirement varies based on the season and university timings, with the maximum demand considered for this calculation. The solar PV panel size (PVsize-req) required is designed for peak summer load, as this period consumes the most energy. In equation (4) [64], peak summer energy consumption is considered for daily energy consumption

(4) $${PV}_{size-req}=\frac{E_{daily}}{{Hr}_{sun}\left(1-{PV}_{systemloss}\right)}=2.511\text{kW}$$

While choosing the solar PV module and inverter, local field weather conditions such as wind, rain, temperature and humidity must be considered, as they affect the performance of the system. Based on research to find the best solar PV module technology suitable for climatic conditions [65,66], ibri's climatic condition closely align with Zone 3. Accordingly, Canadian solar panels and a Sungrow inverter were chosen and adopted for this smart bus stop project. A 550 Wp Canadian Solar Panel is considered for Equation (5) and its main characteristics data [46] is presented in Table 6.

**Table 6:** Canadian Solar Panel characteristics and technical specification data [67].

Model	CS6W-550 MB-AG
Nominal Maximum Power (P_max_)	550 W_p_
Operating Voltage (V_mp_)	41.7 V
Operating Current (I_mp_)	13.20 A
Open Circuit Voltage (V_oc_)	49.6 V
Short circuit current (I_sc_)	14.00 A
Panel Efficiency(η)	21.4 %
Cell Type	Mono-crystalline
Cell Arrangement	144 [2 x (12 x 6)]
Dimensions	2266 ˣ 1134 ˣ 35 mm
Weight	32.2 kg

The number of panels (Npanel-req) needed depends on the size of the Solar PV module [64] calculated in Equation (5).

(5) $$N_{panel-req}=\text{ceil}\left(\frac{{PV}_{size-req}}{P_{\max}\;}\right)=5\;\text{Panels}$$

Based on the calculations, a total of 5 panels are required. The next step is to verify if the size of the solar panels can fit on the bus stop rooftop.

**Fig. 8:**
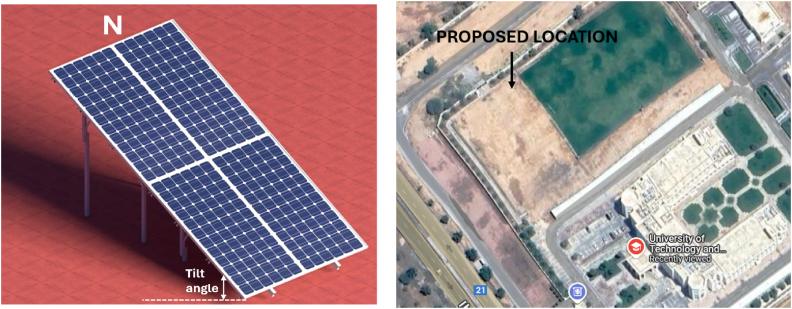
Solar PV illustration and Smart Bus stop proposed location at UTAS-Ibri branch. Source: Google Map.

The bus stop design being considered is rectangular and has a roof size of up to 6.5 m by 4 m. It is planned to be located near by the one of the entrances of the university shown in Fig. 8. Yasser F. Nassar et al. determined the optimum tilt angle to improve the energy yield of a solar PV [[68], [69], [70], [71]]. When considering the installation of a solar panel on the rooftop, the three critical factors come into play: roof space, tilt angle and azimuth angle. The recommended tilt angle is 27.02° and the azimuth angle is set at 180° for maximum power generation [70,71].

The designated small space on top of the bus stops with additional side clearance rooftop is the limitation, does not allow for inter row spacing but can accommodate four panels with a power rating of 550Wp to meet the load requirements. Therefore, a 2.2 kW solar panel is positioned on the bus stop rooftop with a fixed 27.02° tilt angle facing south and a 180° azimuth angle for optimal energy efficiency. Fig. 8 provides a solar PV illustration of the bus stop with the proposed location outlined in this paper. The four panels are connected to the on-grid inverter. If the solar PV system not able to generate sufficient power to meet the bus stop's electricity demand, energy will be sourced from the grid through an on-grid inverter. Based on the data in Table 4, solar PV generation meets the energy needs for most months, except during the peak summer season. During this time and when there is low sunlight, smart bus stop systems will import the energy from the grid.

### 4.2 Sizing of inverter

For an on-grid inverter with an MPPT controller required for the 2.2 kWp solar panel, sizing depends on the solar PV output and peak load. The Maximum DC input power of the inverter is equal to the maximum output power of the solar PV array (PV_array_) [64], as calculated in equation (6),

(6) $${PV}_{array}=N_{panel}*\;P_{\max}$$

Compatibility between Solar PV array and the inverter assessed by considering the derating power (PV_derated_) of the solar PV panel. Mainly Solar PV output power derated due to: Manufacture tolerance of the panel (PV_tol_), Dust deposition (PV_derate-dust_), Temperature effect (PV_derate-temp_), as calculated in equation (9) [72].

The dust derating factor depends on the actual location and level of pollution. Here, if the solar PV is regularly cleaned, the dust derating factor is assumed to be 0.95, which means a 5 % power loss due to dust. If the panel is exposed to high pollution level, the derating factor will be consider lesser than 0.95.

Cell temperature (T_cell-temp_) using NOCT model [73] can be determined using equation (7). Standard data are taken from the solar panel data [67].

(7) $$T_{cell-temp}=T_{avg-temp}+\left(NOCT-T_{STC}\right)\frac{G_{LOCAL}}{G_{STC}}$$

where, $T_{avg-temp}$ is the average temperature in °C; NOCT is the Nominal Module Operating Temperature in°C; $T_{STC}$ and $G_{STC}$ is the under standard test condition module temperature in °C and irradiance in W/m^2^ respectively; $G_{LOCAL}$ is the average irradiance during peak in W/m^2^.

Derated temperature coefficient is calculated in equation (8) [13,49].

(8) $${PV}_{derate-temp}=\left[1+\beta \;\left(T_{cell-temp}-T_{STC}\right)\right]\left(\frac{G_{LOCAL}}{G_{STC}}\right)$$

where, β is the power temperature coefficient in W/°C

(9) $${PV}_{derated}={PV}_{tol}*{PV}_{derate-dust}*{PV}_{derate-temp}$$

This array can be connected to an on-grid inverter with an output rating (Inv_rating_), and it is calculated [72] in equation (10),

(10) $${Inv}_{rating}={PV}_{derated}*{PV}_{array}$$

Therefore, a 2 kW on-grid solar inverter is adequate for the proposed smart bus stop. Data and sizing of Inverter design shown in Table 7. SunGrow residential string inverter SG2K-S is considered, and its key specifications are provided in Table 8.

**Table 7:** Data [48] and the result of sizing an Inverter.

Parameter	Values
Solar PV Peak power (PV_array_)	2.2 kW
Manufactures tolerance of the panel (PV_tol_)	0.95 from data sheet
Average Temperature, Ibri ($T_{avg-temp}$)	37.37 °C
Average irradiance, Ibri ($G_{LOCAL})$	958 W/m^2^
Irradiance under standard test condition ($G_{STC}$)	1000 W/m^2^
Temperature under standard test condition ($T_{STC}$)	25 °C
Cell temperature (T_cell-temp_)	52.698 °C
Derate due to dirt deposition (PV_derate-dust_)	0.95 (Assumed)
Derate due to Temperature (PV_derate-temp_)	0.87
Derating power of the solar PV panel (PV_derated_)	0.78
Rating of on-grid solar inverter (Inv_rating_)	2 kW

**Table 8:** Inverter specification [74].

Model	Maximum PV input voltage (V_max-inv_)	Minimum PV input voltage (V_min-inv_)	Maximum PV input current (I_max-inv_)	Maximum Efficiency	Nominal AC output power (Inv_rating_)
SG2K-S	600V	90V	10A	97.2 %	2000W

#### 4.2.1 Sensitivity analysis on inverter efficiency

**Fig. 9:**
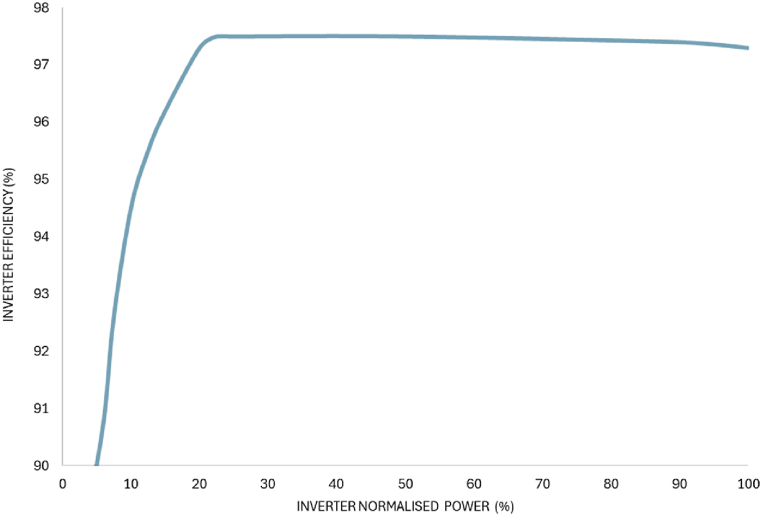
Inverter efficiency variation with percentage of inverter normalised power.

The inverter power output at time (t) depends on the power output from the PV system ${PV}_{array}\left(t\right)$, which depends on the irradiance and derating factor. Under low radiation, inverter produces power with lower efficiency depicted in Fig. 9. Inverter normalised power is the ratio of solar PV output to solar PV rated power. Mathematically inverter normalised power ($P_{inv-NP}$) is estimated [75] by equation (11),

(11) $$P_{inv-NP}\left(t\right)=\frac{{PV}_{array}\left(t\right)}{{PV}_{array}}$$

#### 4.2.2 Mathematical expression for the inverter efficiency

(12) $$\eta_{inv}=\left\{ \begin{array}{c} -347.28{NP}^{2}+135.44NP+84.1,if\;NP=5\%\;to\;20\%\\ -0.0223\;NP+0.975,if\;NP>20\% \end{array}\right.$$

The impact of inverter efficiency variation in Solar PV system output is determined by using inaccuracy in the productivity of the PV solar field (E_ηinv_) [75,76]:

$$E_{\eta inv}=\frac{\sqrt{\sum_{i=1}^{n}{\left(E_{PVexpect}-E_{PVactual}\right)}_{i}^{2}}}{\sum_{i=1}^{n}{E_{PVexpect}}_{i}}$$

Where $E_{PVexpect}$ is the expected power generation of the solar PV array per year, $E_{PVactual}$ is the actual power generation from the solar PV array per year, n is period, here, 8760 h considered. Table 9 shows the number of hours data of the solar PV array output in terms of inverter efficiency and average estimated error. From the sensitivity analysis, inverter efficiency variation impact is very small on the solar PV system output, as the average estimated error is 0.017 %.

**Table 9:** Sensitivity Analysis on Inverter efficiency.

Parameter	Values
Number of hours electricity produced at inverter efficiency of more than 97.5 %	3896
Number of hours electricity produced at inverter efficiency of less then 97.5 %–90 %	400
Number of hours, zero electricity produced	4464
Number of hours (n)	8760
average estimated error	0.017 %

### 4.3 Voltage matching between the array voltage and the inverter input voltage

The actual input voltage to the inverter form the solar PV must be within the inverter's specified voltage range for efficient operation [77].

The maximum input voltage of an inverter depends on the open circuit voltage (Voc) of the solar PV. At low temperatures, the open circuit voltage increases. This increased voltage should not exceed the inverter's maximum input voltage.

The minimum input voltage of an inverter depends on the maximum power point voltage (Vmp). As the temperature increases, Vmp decreases. This reduced Vmp does not drop below the inverter's minimum input voltage. The Canadian solar panel temperature characteristics are shown in Table 10 to verify the voltage range of the inverter.

**Table 10:** Canadian solar Panel temperature characteristics [67].

Power Temperature Coefficient (β)	Temperature Coefficient (V_oc-tempcoeff_)	Temperature Coefficient (I_sc-tempcoeff_)	Nominal operating cell Temperature (NOCT)	Operating Temperature (t_panel-max_)
−0.34 %/°C	−0.26 %/°C	0.05 %/°C	41 ± 3 °C	85 °C

Minimum voltage allowed to the inverter (Vmp-inv) is calculated using equations (13), (14)

(13) $$V_{mp-tmax}=V_{mp}+\left(\left(t_{panel-\max}-t_{STC}\right)*V_{mp-tempcoeff}\right)$$

Where, $V_{mp-tmax}$ is MPP voltage at a maximum temperature in volts, $V_{mp}$ is MPP voltage at STC in volts, $t_{panel-\max}$ is Maximum cell operating temperature in °C, $t_{STC}$ is cell temperature at STC in °C, $V_{mp-tempcoeff}\text{,}$ Voltage temperature (Vmp) coefficient in volts per °C.

Minimum input voltage allowed to the inverter (V_mp-inv_) in volts by considering maximum voltage drop in the cables ($V_{drop-cable}$),

(14) $$V_{mp-inv}=V_{mp-tmax}*\left(1-V_{drop-cable}\right)$$

Therefore, Minimum number of panels (Nmin-module) in a string to maintain minimum inverter input voltage is,

(15) $$N_{\min-module}=\text{ceil}\left(\frac{V_{\min-inv}}{V_{mp-inv}}\right)$$

Where, $V_{\min-inv}$ is the minimum inverter input voltage in volts.

Normally, lowest temperature in Oman is around 13 °C.

Maximum open circuit voltage at Minimum temperature (V_oc-tmin_) is,

(16) $$V_{oc-tmin}=V_{oc}+\left(\left(t_{\min}-t_{STC}\right)*V_{oc-tempcoeff}\right)$$

Where, $V_{oc-tmin}$ is open circuit voltage at minimum cell temperature in volts, $V_{oc}$ is open circuit voltage at STC in volts, $V_{oc-tempcoeff}$ is open circuit voltage temperature coefficient in -V/°C, $t_{\min}$ is minimum daily temperature in °C, $t_{STC}$ is cell temperature at STC in °C.

Maximum number of panels (N_max-module_) in a string to maintain Maximum inverter input voltage (V_max-inv_) is,

(17) $$N_{\max-module}=\text{ceil}\left(\frac{V_{\max-inv}}{V_{oc-tmin}}\right)$$

Where, $V_{\max-inv}$ is the maximum voltage allowed by the inverter in volts.

The Minimum and Maximum number of panels in a string is calculated using equations (13), (14), (15), (16), (17) to match the array voltage to the inverter voltage range is calculated and result is shown in Table 11. A proposed smart bus stop PV string must consist of between 3 and 12 panels in a string. According to the calculation, four 550Wp panels are connected in series to form a string.

**Table 11:** Voltage match results.

Parameter	Values
Maximum power point voltage at Maximum temperature (V_mp-tmax_)	32.7V
Maximum voltage drop in the cables (V_drop-cable_)	3 % assumed
Minimum input voltage allowed to the inverter (V_mp-inv_)	31.719V
Minimum number of panels (N_min-module_) in a string to maintain V_min-inv_	∼3 Panels
Oman lowest temperature (t_min_)	∼20 °C
Maximum open circuit voltage at Minimum temperature (V_oc-tmin_)	52.52 V
Maximum number of panels (N_max-module_) in a string to maintain V_max-inv_	∼12 Panels

## 5 Performance analysis, economic analysis and environmental analysis of solar PV system, Ibri

### 5.1 Performance analysis

#### 5.1.1 Solar array output

The Solar PV output (1) is modified due to various factors such as: Manufacture tolerance of the panel and dust deposition presented in (16). The array output (P_array_) is further modified when it is converted to AC energy due to cable loss, inverter efficiency and AC system loss. The total AC energy output (Pac) per day [64] is calculated using equation (18). The results of solar array output for smart bus stop, Ibri, are shown in Table 12.

(18) $$P_{derated}={PV}_{derated}*P_{\max}$$

(19) $$P_{array}=P_{derated}*N_{panel}*{Hr}_{sun}$$

(20) $$P_{ac}=P_{array}*\left(1-P_{cable-loss}\right)*P_{inv-eff}*\left(1-P_{AC\;losses}\right)$$

(21) $$E_{ac}=P_{ac}*Number\;of\;Days$$

**Table 12:** Solar array output, Ibri.

Parameter	Values
Derating factor of the solar PV array (PV_derated_)	0.82
Panel derated power (P_derated_)	451W
DC Energy output from array per day (P_array_)	14.161 kWh
Cable loss ($P_{cable-loss}$)	3 % (Assumed)
AC system loss $\left(P_{AC\;losses}\right)$	1 %(Assumed)
Total AC Energy output per day (Pac)	13.354 kWh
Annual energy form solar PV array ($E_{AC})$	4840 kWh

#### 5.1.2 Specific energy yield

It is a measure the annual energy yield ($E_{Specific-yield}$) relative to the total installed capacity, calculated [78] using equation (22).

(22) $$E_{Specific-yield}=\frac{E_{AC}}{{PV}_{\max}*N_{panel}}=2200\;kWh/kWp$$

#### 5.1.3 Performance ratio

Performance ratio (PR) is used to measure the performance of the unit by considering losses in the system and calculated [78] using equation (23),

(23) $$\mathrm{PR}=\frac{E_{AC}}{{Hr}_{sun}*N_{panel}*P_{\max}*365}=0.77$$

#### 5.1.4 DC capacity factor

DC Capacity factor (CF) measures the solar energy generation per day relative to the maximum capacity of the solar PV system, calculated [78] using equation (24),

(24) $$CF=\frac{P_{array}}{{PV}_{\max}*24}=0.27$$

### 5.2 Levelized cost of electricity of the solar PV system

The economic feasibility of solar electricity is analysed by the levelized cost of electricity (LCOE) of solar PV system [79,80]. The basic formula is shown in (25).

(25) $$LCOE=\frac{Total\;life\;time\;cost\;\left(C_{t}\right)}{Total\;life\;time\;energy\;production\left(E_{t}\right)}=\frac{\sum_{t=0}^{N}\frac{I_{t}+O_{t}+M_{t}+F_{t}+R_{t}}{{\left(1+d_{r}\right)}^{t}}}{\sum_{t=0}^{N}\frac{E_{array-year}{\left(1-d\right)}^{t}}{{\left(1+d_{r}\right)}^{t}}}$$

Total lifetime cost and energy production is assessed using discount rate. This variable is critical variable, used to discount future cash flows back to their present value, it reflects the project's capital cost.

Solar PV cost: four 550 Wp Canadian solar panels were purchased at a cost of around $2400, including import taxes and GST. The current market rate for solar PV is approximately $0.28 per watt.

Inverter cost: An on-grid inverter was purchased at a cost of $1700, including all taxes. The solar PV panels and inverter constitute the major portion of the investment. Installation labour and miscellaneous costs add up to $220.

Investment cost (It): It includes solar PV cost, Inverter cost and Installation Labour and Miscellaneous cost.

Operation cost (Ot): This cost includes general monitoring using equipment and software. It covers electricity needed for monitoring equipment, communication systems, and auxiliary components. A specialized application is used to monitor solar PV performance from both mobile and desktop devices. Operation costs are 0.5 % of the total capital cost.

Maintenance cost (Mt): Regular water cleaning is recommended for 2.2 kW solar panel. Maintenance cost includes the cleaning labour cost, inspection cost and service cost. A cost of 1 % of installation cost per kW is considered for maintenance.

Replacement cost (Rt): A on grid inverter is used in this solar PV system to import power from grid when solar PV power it is not enough to meet the demand of smart bus stop. The typical lifespan of an inverter is 10 years, and replacement costs are considered every 10 years over the project's total lifespan, amounting to 10 % of the total investment cost.

Financing cost (Ft): Currently, there is no specific policy to provide financial support such as initial loans, lower interest rate for residential rooftop solar PV projects. Therefore, financing cost is solely based on the annual discount rate, which is 3 % in this case.

Based on the calculations above, the LCOE of solar PV in UTAS Ibri branch is determined and required data tabulated in Table 13.

**Table 13:** LCOE Data of Solar powered smart bus stop in UTAS-Ibri.

Parameter	Values
Total Solar PV cost	$2400
Inverter cost	$1700
Installation Labor cost and Miscellaneous cost	$220.08
Total Investment cost (It)	$4320.08
Operation cost (O_t_)	0.5 % of total Installation cost
Maintenance cost (M_t_)	$43.2/kW+$3 for every year
Replacement cost (R_t_)	For every 10th year 10 % of the total investment cost
Discount (r)	3 %
Annual AC energy output (S_t_)	12.29 kWh
Degradation rate (dr)	0.5 %
Lifetime of the project (N)	25 years

### 5.3 Environmental benefit of the solar PV system

**Fig. 10:**
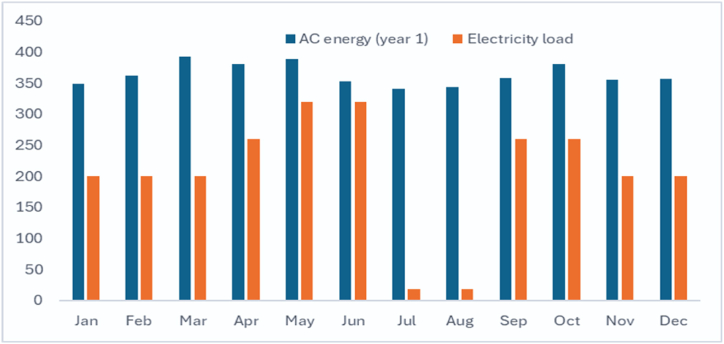
Monthly solar PV power generation (kWh) and power consumption(kWh) of smart bus stop at UTAS-Ibri branch.

The smart bus stop load is primarily met by solar PV generation. Fig. 10 shows the monthly solar power generation and power consumption of the smart bus stop. During vacations and weekends, excess solar power is exported to the grid and mix with conventional power generation, and thereby reducing the CO_2_ emission. In Oman, natural gas contributes the maximum share to electrical power generation [3]. The cost of avoided carbon emission due to the solar PV generation system [81,82] can be calculated in terms of social cost of carbon ($C_{CO2\;avoided}$) by the following equation (26).

(26) $$C_{CO2\;avoided}={EF}_{CO2}\times E_{AC}\times \emptyset_{CO2\;}$$

Where, $C_{CO2\;avoided}$ is cost of avoided carbon emission due to the solar PV generation in $ per year, ${EF}_{CO2}$ represents the CO_2_ emission factor of the electric power generation system (kg CO_2_/kWh), $\emptyset_{CO2\;}$ represents the carbon social cost ($/ton CO_2_), which may be considered as $ 70/ton CO_2_.

Table 14 shows the environmental benefit of the solar PV system per year. The solar-powered smart bus stop located at Ibri results in an environmental benefit of approximately $146.7 per year based on a social cost of carbon.

**Table 14:** Environmental benefit calculation of Solar powered smart bus stop in UTAS-Ibri.

Parameter	Values
CO_2_ emission factor of the electric power generation from Natural gas system (kg CO_2_/kWh) (${EF}_{CO2}$)	0.433 kg CO_2_/kWh
Annual energy form solar PV array ($E_{AC})$	4840 kWh
carbon social cost ($\emptyset_{CO2\;}$)	70 $/ton CO_2_
Environmental benefit in terms of social carbon cost (${SCC}_{benefit})$	$146.7 per year

## 6 Results and discussion

### 6.1 Performance analysis, economic analysis and environmental analysis of solar powered smart bus stop at various UTAS branches

#### 6.1.1 Performance analysis

To assess the efficiency of solar photovoltaic systems across various UTAS branches, it is assumed that the same set up of Canadian solar panels, cables, room sizes, loads, and inverters are virtually used in the smart bus stop at all UTAS branches. The data required to evaluate the solar array's performance has been collected from System Advisor Model (SAM).

During the peak summer months from May to August spanning 120 days as depicted in Table 15 the performance of solar PV panels at various branches is detailed. At high temperature, the glass over of the solar PV panels tends to absorb more heat, which leads to reduces the output. The dust derating factor is decided by the impact and frequency of the sandstorms in the corresponding areas. Desert regions like Ibra, Nizwa, and Ibri are frequently experienced sandstorms leading to more dust accumulation on solar panels, which reduces their performance. Coastal regions like Al Musannah, Shinas, Muscat and Sur have moderate amount of dust and salt deposition on solar panels reduces their output. During the summer, dust deposition on solar panel located in Salalah is lesser compared to other branches due to Khareef season begins in Salalah. As a result, solar panel in Salalah has lower dust derating factor and Ibri has higher dust derating factor.

**Table 15:** Performance analysis of solar PV system at various UTAS-Branches during summer.

UTAS branches	T_avg-temp_ (approx.)	PV_derate-temp_	PV_derate-dirt_	PV_derated_	P_derated_ (W)	${Hr}_{sun}$	P_array_ (kW)	P_ac_ (kW)	E_AC_ (kWh/Ns^a^)	CF	E_specific-yield_ (kWh/kW)	PR
Muscat	40.38	0.86	0.85	0.70	383.15	9.09	13.93	13.14	1576.49	0.26	716.59	0.66
Salalah	34.52	0.88	0.92	0.77	424.28	7.32	12.42	11.72	1405.80	0.24	639.00	0.73
Nizwa	42.02	0.86	0.80	0.65	358.28	8.86	12.70	11.97	1436.87	0.24	653.12	0.61
Ibra	40.55	0.86	0.80	0.66	360.37	8.89	12.81	12.08	1450.14	0.24	659.16	0.62
Shinas	40.28	0.86	0.85	0.70	383.30	8.68	13.31	12.55	1505.98	0.25	684.54	0.66
Sur	41.22	0.86	0.85	0.69	381.88	8.88	13.56	12.79	1534.98	0.26	697.72	0.65
Ibri	42.69	0.85	0.80	0.65	357.33	9.26	13.24	12.48	1497.75	0.25	680.79	0.61
Al Mussanah	41.03	0.86	0.85	0.69	382.17	8.99	13.74	12.96	1555.16	0.26	706.89	0.66

a Ns: Number of summer days.

During the summer in Oman, Salalah experiences the Khareef season which is like monsoon, with frequent mist, light rain and fewer hours of sunshine, results lesser solar energy production than other regions. While Ibri has higher irradiation than other places, its solar PV energy generation is reduced due to the temperature derating and frequent sandstorms. According to derating factor, Salalah has a lower than other branches, however lesser sun hours result in lesser solar energy compared to other branches. The Coastal area's solar PV generate more solar energy compared to other areas. Among all the branches Muscat branch solar PV generates more energy during summer.

The derating factor caused by dust can be further minimized through regular cleaning. The smart solar PV bus stop, which has four panels, does not require advanced technology for maintenance. since it's situated on a university campus where the maintenance staff can regularly clean the panels. As a result, a 5 % reduction in power output due to dust accumulation is assumed. Consistent cleaning leads to enhanced solar energy generation, as shown in Table 16. This results in a significant increase in solar energy yield at various UTAS branches, particularly in Ibri, and an improvement in the performance ratio.

**Table 16:** Performance analysis with regular cleaning of solar PV system at various UTAS-Branches during summer.

UTAS branches	T_avg-temp_ (approx.)	PV_derate-temp_	PV_derate-dirt_	PV_derated_	P_derated_ (W)	${Hr}_{sun}$	P_array_ (kW)	P_ac_ (kW)	E_AC_ (kWh/Ns^a^)	CF	E_specific-yield_ (kWh/kW)	PR
Muscat	40.38	0.86	0.95	0.78	428.23	9.09	15.57	14.68	1761.96	0.29	800.89	0.73
Salalah	34.52	0.88	0.95	0.80	438.12	7.32	12.83	12.10	1451.64	0.24	659.84	0.75
Nizwa	42.02	0.86	0.95	0.77	425.46	8.86	15.08	14.22	1706.28	0.29	775.58	0.73
Ibra	40.55	0.86	0.95	0.78	427.94	8.89	15.22	14.35	1722.04	0.29	782.75	0.73
Shinas	40.28	0.86	0.95	0.78	428.40	8.68	14.87	14.03	1683.16	0.28	765.07	0.73
Sur	41.22	0.86	0.95	0.78	426.81	8.88	15.16	14.30	1715.56	0.29	779.80	0.73
Ibri	42.69	0.85	0.95	0.77	424.33	9.26	15.72	14.82	1778.58	0.30	808.44	0.73
Al Mussanah	41.03	0.86	0.95	0.78	427.13	8.99	15.36	14.48	1738.12	0.29	790.05	0.73

a Ns: Number of summer days.

The temperature related derating factor is increased due to the reduced temperature during the non-summer period, which increases the solar output. However, during this period, the solar array output per day is reduced due to the less sun hours and lower solar irradiance, as shown in Table 17. All branches generate nearly equal amount of solar energy output. The performance ratio of solar PV systems in all branches are improved due to the increased derating factor.

**Table 17:** Performance analysis of solar PV system at various UTAS-Branches during non-summer.

UTAS branches	T_avg-temp_ (approx.)	PV_derate-temp_	PV_derate-dirt_	PV_derated_	P_derated_ (W)	${Hr}_{sun}$	P_array_ (kW)	P_ac_ (kW)	E_AC_ (kWh/Nns^a^)	CF	E_specific-yield_ (kWh/kW)	PR
Muscat	29.06	0.90	0.94	0.80	442.62	7.11	12.59	11.87	2908.36	0.24	1321.98	0.76
Salalah	26.88	0.91	0.97	0.84	460.51	6.56	12.08	11.40	2791.79	0.23	1269.00	0.79
Nizwa	29.59	0.90	0.90	0.77	422.94	7.11	12.03	11.34	2779.03	0.23	1263.19	0.73
Ibra	28.13	0.90	0.90	0.77	425.27	7.18	12.21	11.52	2821.88	0.23	1282.67	0.73
Shinas	28.91	0.90	0.94	0.81	442.87	7.01	12.42	11.71	2869.07	0.24	1304.12	0.76
Sur	29.83	0.90	0.94	0.80	441.34	7.05	12.45	11.74	2875.43	0.24	1307.02	0.76
Ibri	28.11	0.90	0.90	0.77	425.31	7.23	12.30	11.60	2841.74	0.23	1291.70	0.73
Al Mussanah	29.06	0.90	0.94	0.80	442.62	7.03	12.45	11.74	2875.63	0.24	1307.11	0.76

a Nns: Number of non-summer days.

During the non-summer period, dust accumulation is lower compared to the summer period. Consequently, 3 % reduction factor is assumed for dirt in this analysis. Regular cleaning enhances the energy yield of the solar PV system, as indicated in Table 18.

**Table 18:** Performance analysis with regular cleaning of solar PV system at various UTAS-Branches during non-summer.

UTAS branches	T_avg-temp_ (approx.)	PV_derate-temp_	PV_derate-dirt_	PV_derated_	P_derated_ (W)	${Hr}_{sun}$	P_array_ (kW)	P_ac_ (kW)	E_AC_ (kWh/Nns^a^)	CF	E_specific-yield_ (kWh/kW)	PR
Muscat	29.06	0.90	0.97	0.83	456.75	7.11	12.99	12.25	3001.18	0.25	1364.17	0.78
Salalah	26.88	0.91	0.97	0.84	460.51	6.56	12.08	11.40	2791.79	0.23	1269.00	0.79
Nizwa	29.59	0.90	0.97	0.83	455.84	7.11	12.96	12.23	2995.17	0.25	1361.44	0.78
Ibra	28.13	0.90	0.97	0.83	458.35	7.18	13.16	12.41	3041.36	0.25	1382.43	0.79
Shinas	28.91	0.90	0.97	0.83	457.01	7.01	12.81	12.08	2960.64	0.24	1345.75	0.78
Sur	29.83	0.90	0.97	0.83	455.42	7.05	12.84	12.11	2967.20	0.24	1348.73	0.78
Ibri	28.11	0.90	0.97	0.83	458.39	7.23	13.26	12.50	3062.77	0.25	1392.17	0.79
Al Mussanah	29.06	0.90	0.97	0.83	456.75	7.03	12.84	12.11	2967.41	0.24	1348.82	0.78

a Nns: Number of non-summer days.

**Fig. 11:**
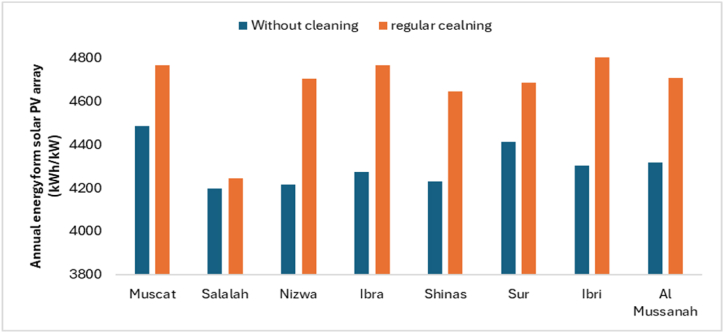
Comparison of Annual energy yield of solar PV systems with cleaning and without cleaning located at various UTAS branches.

The total energy yield throughout the year fluctuates depending on factors such as sun hours, temperature, and dust accumulation. Fig. 11 illustrates the annual energy yield at various UTAS branch locations, comparing scenarios with regular cleaning versus without cleaning of solar PV panel.

**Fig. 12:**
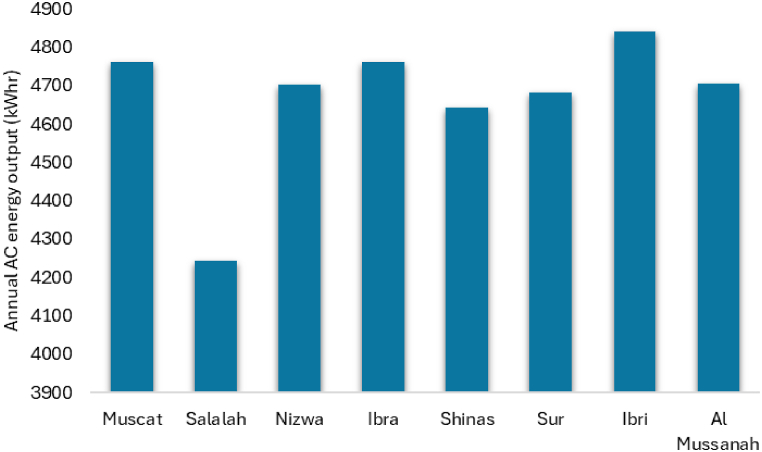
Annual energy output of solar PV systems located at various UTAs branches.

The total AC energy output of the solar PV systems located at various branches for the first year is presented in Fig. 12. Depending on the location and environmental conditions, AC energy generation from solar PV systems of the same capacity ranges from 4243.44 kWh to 4841.34 kWh. Ibri has highest AC energy generation due to long sun hours.

#### 6.1.2 Economic analysis

**Fig. 13:**
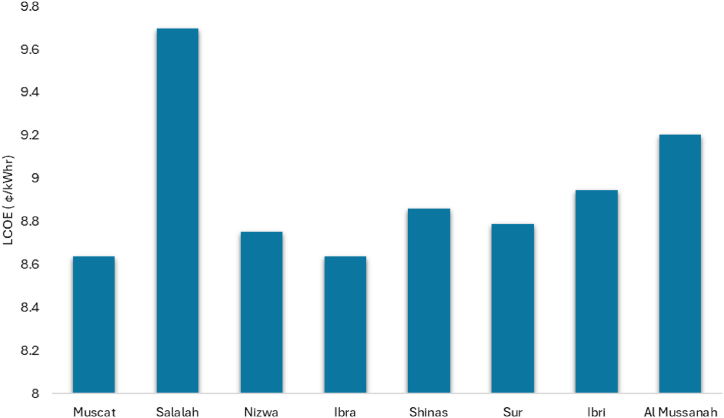
LCOE of solar PV systems located at various UTAs branches.

The LCOE of solar PV systems at various UTAS branches is calculated using Equation (25). The LCOE ranges from 8.5 ¢/kWh to 9.7¢/kWh. Due to local weather conditions, Salalah has a higher LCOE of solar PV system than other branches. Except for the Salalah branch, the LCOE of solar PV systems at all other branches nearly equal LCOE, as shown in Fig. 13.

#### 6.1.3 Environmental benefit

**Fig. 14:**
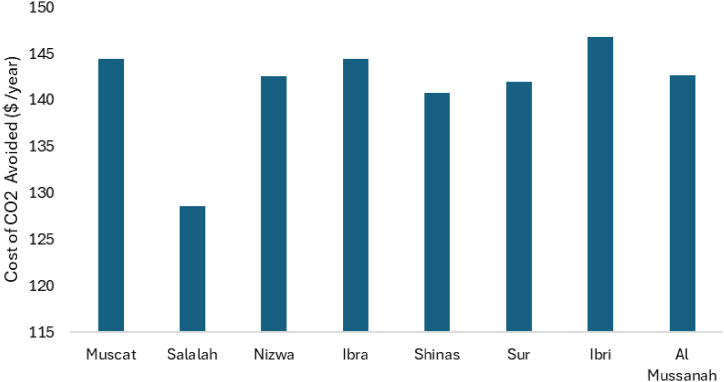
**A**voided carbon emission cost of solar PV systems located at various UTAs branches.

The environmental benefit of the solar-powered smart bus stop located at various locations are calculated using Equation (26) and shown in Fig. 14. The environmental benefit, in terms of social carbon cost (${SCC}_{benefit})$, is calculated for a smart bus stop load. Since solar PV systems produce no carbon emissions, the avoided carbon emission cost varies from 128.16 $ per year to 146.74 $ per year.

### 6.2 Design and analysis of solar PV system for smart bus stop using SAM software

The System Advisory Model (SAM) is specifically designed software for modelling and analysing the performance of both on grid and off-grid solar PV projects. The System Advisory Model (SAM) is a robust tool for analysing the renewable energy systems, renewable energy potential, particularly solar PV and solar thermal systems [83,84]. This software is used to model, analyze the solar PV systems for the smart bus stop, and its results are validated with the analytical approach results. Design procedure, parameter setting and device selection of the solar PV system using SAM is shown in Table 19.

**Table 19:** Solar PV system design flow for smart bus stop using SAM.

Design Flow	Selection of Devices/Set the parameters
**Input Data for Location and Resources:** Set the location of the university. Download weather file data and calculate the annual averages of solar irradiance for that location.	Refer Table 3
**Panel selection** Select the suitable panel from the panel database	CS6W-550 MB-AG
**Inverter selection** Select the suitable inverter from the inverter database	SG2K-S
**Design a System:** Set the number of inverters. Configure the number of panels per string in the subarray Set tracking and orientation.	Number of Inverters: 1 Panels per string in sub array: 1 Number of Panels in sub array: 4 Tilt angle = calculated [74] Azimuth = 180° Tracking = Fixed
**Calculate losses:** Irradiance loss due to soiling, DC loss, AC loss, Transformer loss, Transmission loss Set the percentage of soiling loss	Soiling losses = 5 % DC wiring loss = 3 % AC loss = 1 %
Set grid limit	It is set to export the power when it is not utilised.
Set annual Degradation rate	0.5 %
Installation cost Set investment cost: Set operation and maintenance cost Set inflation rate, real discount rate Set sales tax and incentives	Investment cost = $4320 Operation and maintenance cost: 43.2 $/kW-yr Indirect capital cost, inflation rate, real discount rate = $0 Sales Tax = $0
Upload Electricity Tariff	As per NAMA Tariff [85]
Upload smart bus stop load	Table 4
Simulate the software and collect the summary report	

The following assumptions are considered to facilitate the analysis:

The NOCT method proposed by SAM simulator was followed for temperature correction of the solar module.

The optimum tilt angle is calculated based on the method presented by Alsadi Samer et al. [70].

Weather data is taken from Solar resource library from SAM software.

The same design parameters and components are used for all locations, with only the tilt angle adjusted based on the calculations.

## 7 Validation of analytical method results with SAM results

### 7.1 Annual energy yield

**Fig. 15:**
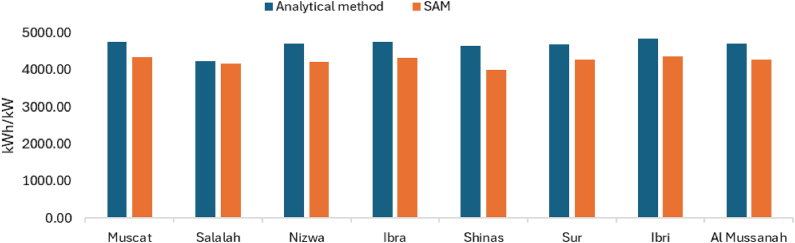
Solar PV annual energy output of various UTAS branches.

The annual energy output of the solar PV, as calculated using Equation (21), and as obtained from SAM, is shown for various UTAS branches in Fig. 15. Both methods show that Ibri branch provide the maximum solar energy output, while Salalah register the minimum solar energy output.

### 7.2 Performance ratio

**Fig. 16:**
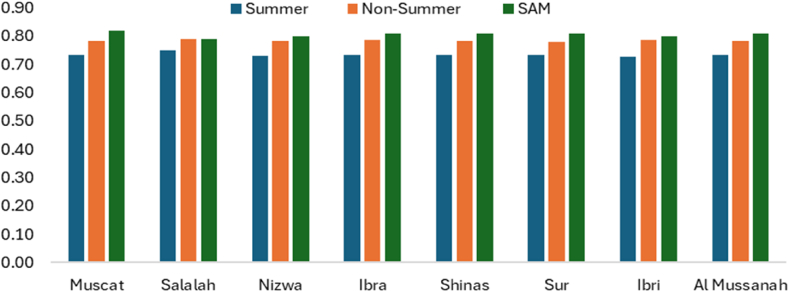
Solar PV Performance ratio of various UTAS branches.

Fig. 16 shows the performance ratio during the summer period, the non-summer period, and the SAM results. SAM considered a whole year to calculate the performance ratio. The degradation rate due to dirt is considered constant, assuming regular cleaning throughout the year and calculated performance ratio for summer period and non-summer period using equation (23). During the summer, the performance ratio is around 0.73 due to temperature effects, while during the non-summer period, it is approximately 0.80. The SAM results also proves that the performance ratio is around 0.8 for all branches.

### 7.3 DC capacity factor

**Fig. 17:**
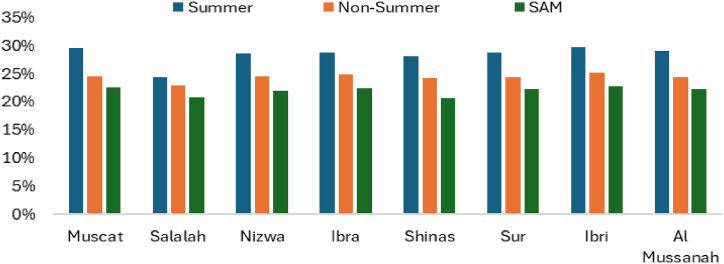
Solar PV DC Capacity factor for various UTAS branches.

Fig. 17 displays the DC Capacity factor is calculated using equation (24) for the summer period and non-summer period, along with the SAM results. During the summer, the DC capacity factor is higher compared to the non-summer period due to increased number of sun hours.

### 7.4 LCOE

**Fig. 18:**
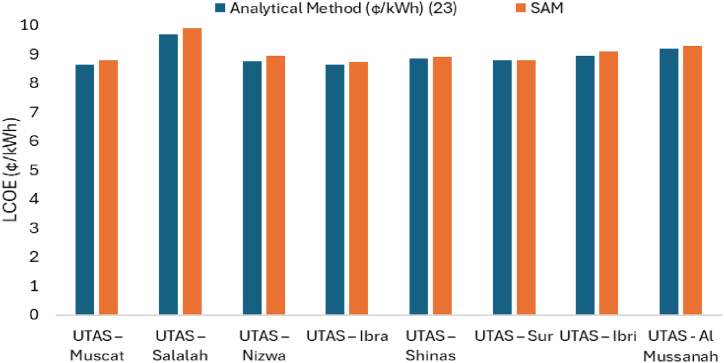
LCOE of solar PV for various UTAS branches.

Fig. 18 displays the LCOE based on calculations using equation (25) and the SAM result. Both results show nearly the same values. All UTAS branches have a LCOE of around 8 ¢/kWh except for Salalah, it has the highest LCOE 11.68¢/kWh.

Electricity generation in Oman mostly depends on natural gas, with the estimated Levelized Cost of Electricity (LCOE) ranging from $30 to $40 per megawatt hour, as stated in a report by the International Energy Agency [86]. On the other hand, solar photovoltaic systems in various location of Oman have an LCOE of around $80 per megawatt hour from the study. While natural gas provides a cost efficient and reliable energy source, concerns about its environmental impact are driving the adoption of alternative energy solutions. Despite solar PV currently having a higher LCOE, advancements in technology and market trends are projected to reduce these costs, making solar PV a more sustainable and competitive choice for the future. This case study offers an information on solar PV power generation on rooftops in optimal locations in Oman, considering various factors. The northern regions of Oman are the most suitable locations to install the solar PV systems. In the northern regions Ibri is the best locations for rooftop solar PV projects compared to other locations. However, other locations are also competitively suitable for solar PV projects. This case study highlights that solar PV is a choice for sustainable energy and aligns with Omans renewable energy goals of Oman Vision 2040. However, the lack of a policy in Omans's energy sector hinders the promotion of rooftop solar PV.

## 8 Impact of the smart bus stop research case study results on economy, environment and society

The smart bus stop is proposed to be located on the university campus, serves as an educational model for sustainable energy solution in smart city infrastructure. It practically demonstrates the feasibility and benefits of renewable energy solutions in inspiring way, which motivating students to do more future projects in the field of renewable energy. The implementation of this project in various locations in Oman brings the following impact on economy, environment and society [87].

• It encourages the public to use the public transport and adopt energy saving practices, which, in turn reduces the carbon foot print, decreases the fossil fuel usage and lowers the operating costs.
• Energy-efficient smart loads minimize energy wastage and further reduces the maintenance cost.
• Solar-powered smart systems create public awareness about using the renewable energy and energy efficient systems in their homes and commercial places, which, in turn increases the market value of Solar PV power.
• The growth of the Solar PV power market creates more employment opportunities and fosters new startups.
• It provides more research opportunities for the young minds of Oman.
• It promotes to achieve diversification in the power generation sector.

## 9 Attractive solar PV strategies for Oman

Rooftop solar PV systems have been successfully implemented in many countries and provide good revenue to both the owners and the government due to attractive and supportive policies and regulations. Hence, a strong policy framework and regulations are important to make the public awareness and to establish the rooftop solar PV system.

Table 20 highlights the attractive policies that have been implemented over the years in the world's leading solar PV countries. A review of policies implemented by leading solar PV countries shows that many initially promoted solar power by introducing fixed FiT, which effectively supported the early adoption of solar systems. As the cost of solar PV systems decreased, these policies were updated accordingly. Subsidy programs for solar PV installation provided financial support to homeowners, driving greater participation in solar initiatives. Some countries have implemented a solar PV business model for low-income groups, allowing people to purchase solar PV system through low interest loans and earn money by selling the electricity through net metering [109]. Also, the government initiate and invest in research and development and launches public awareness programs. Today, the top rooftop solar nations are advancing to the next level by transforming entire cities into green energy or zero-energy buildings, supported by government financial assistance and subsidies. In Oman, a greater number of Oman Nationals are willing to use residential solar PV, but they are not aware of solar energy programs [32]. Therefore, suggesting the following strategies based on successful strategies, best practices from other countries, and their policies and regulation can enhance the promotion of rooftop solar PV systems and to support to frame the policy.

• Design a dynamic FiT [110] to export solar power to the grid, allowing homeowners can sell their excess solar power through net metering at a dynamic tariff.
• Allocating subsides to install rooftop solar PV systems in home reduces the financial burden on homeowners.
• Implementing a solar PV business model for economically weaker sections to help financially by allowing them to sell the solar power.
• Introducing an incentive to compensate for solar electricity as it is expensive compared to existing electricity tariff makes it economically sustainable.
• Allocate special funding and launch a public awareness program on residential solar PV.

**Table 20:** Review of supportive rooftop solar PV policies.

Country	Policies Followed	Findings	Reference
Germany	Feed in Tariff (FiT)	Series of legalization has been applied for most effective feed in tariff.	[88]
	Market Incentive Program	Bank provides extra funding for small size renewable powered installation.	[89,90]
	Hauswende policy	Low-interest rate loans to renovate their buildings to install solar panels.	[91]
	Solar energy Auction	Instead of FiT, solar PV developers can participate in competitive auctions to sell their solar generated electricity.	[92]
China	Renewable Energy law	In 2005, government framed renewable energy law under this the following mechanism established: 1. Ensures the market scale and direct investment of renewable energy by protecting strong policy. 2. Framed purchase policies to grid companies to purchase all renewable electricity. 3. Renewable energy producers favored feed in tariff system. 4. Introduced a cost sharing mechanism to sell renewable electricity to the utilities and end users. 5. The renewable energy special fund to support renewable energy research projects.	[93,94]
	The brightness program and Township Electrification Program	In 1996, The brightness program provided 100W per person on daily basis through solar PV and wind power to the un electrified area. In 2002 Township electrification program provided standalone solar PV to the un electrified towns.	[94]
	Solar subsidy Program	Solar Roof Program: In 2009, rooftop subsidy program provides a subsidy for less than 50 kW rooftop system includes on grid inverter, battery and solar PV. Golden sun demonstration Program: In 2009, this program provides 50 % subsidy for the on grid solar PV installation cost and 70 % for the off grid solar PV installation cost for more than 300 kW system size.	[95]
	Poverty Alleviation Program	This program provides financial help to poor regions by installing rooftop solar PV on their homes and community building and sell the solar PV electricity to the grid and the revenue is shared with the participating homeowners. For installation of solar PV, they can get subsidy 3000 yuan from Government and 70 % loan from bank.	[96]
	Feed in Tariff	In 2011, national wide stable and fixed FiT policy provides the FiT price is higher than the concession bidding process rate. This FiT rate is vary based on the location and type of renewable energy.	[97]
	Free grid connection services	The state grid corporation of China provides the free connection services such as technological assistance, equipment testing, grid integration plan for the installed capacities of less than 6 MW to help struggling domestic solar PV industry.	[93]
USA	Energy Policy Act 2005	In 2005, this policy provided a 30 % investment tax credit for PV system investment. This act was complemented by accelerated depreciation, which was approximately 26 % to the tax benefit. It reduces the system cost approximately 56 % over six period of many investors.	[97,98]
	Solar America Initiative	In 2007, under this scheme 13.7 million USD sponsored to eleven university led advanced solar PV projects in manufacturing process and products.	[99]
	Sun shot initiative	In 2011, this program helped to reduce the cost of solar installation and supported the growth of solar energy as a viable mainstream power source.	[100]
	Greenhouse gas reduction fund - Solar for all program	A part of this fund is aimed to support the under privileged community, low income group to benefit from distributed solar energy.	[101]
	State incentives	Each state have different incentives, financing options like state tax credits, performance based incentives and solar renewable energy certificates which solar electricity provider can sell.	[102]
	Feed in Tariff	Each state has different feed in Tariff. Through net metering policy, home owners can sell their extra solar electricity.	[103]
Japan	Feed in tariff	In 2012 new FiT scheme was introduced for ten to twenty years contract, it is fixed and above market rate.	[104]
	National subsidy program	The government introduced in 1994 for residential installation of solar PV. This subsidy was covered 50 % of the installation cost for installed capacity maximum up to 5 kW.	[105]
	Zero energy houses	One way for zero energy houses is installing solar PV systems on their building. Building owners receive subsidies under this scheme to construct and renovate their houses to Zero energy houses	[106]
India	Rooftop Solar Programme Phase-II subsidy	It offers financial incentives for the rooftop solar PV installation. Homeowners can receive up to 40 % Capital subsidy for system up to 3 kW and 20 % subsidy for system between 3 kW and 10 kW.	[107]
	Net metering policy	This scheme provides to the homeowners can export the solar electricity to the grid and receive credits on their electricity bills.	[108]

## 10 Conclusion

This study assessed the suitable region in Oman for installing residential rooftop solar PV projects to support Oman vision 2040's renewable energy goal. The key factors considered to find the best regions include solar PV irradiation level, temperature coefficient, humidity level, dust accumulation and population density. The initial qualitative assessment revealed that mountain regions and central part of Oman have high temperature coefficient, dust accumulation and sparse population densities making it less feasible, for installing solar PV projects. With the exception of humidity, the northern region and Dhofar region exhibit favourable conditions in terms of all other factors. A case study of 2.2kWp rooftop solar PV system for a smart bus stop on UTAS- Ibri branch was designed and, its performance, LCOE and environment benefit were compared with that of other UTAS branches in various locations in Northern region and Dhofar region. The findings showed that performance ratio at all locations decreases during the summer compared to non-summer periods due to high temperature experienced in Oman. Ibri is the top location in Oman suitable for solar PV projects. Their annual energy output is further increased by regular cleaning of the solar PV panels. Other locations in the Northern region also show a good annual energy yield from solar PV. The performance analysis is verified with SAM software, which shows almost the same results as the analytical method. Finally, the rooftop policies and regulation of successful countries are discussed and strategies to promote rooftop solar PV in Oman were suggested, such as net metering, FiT, subsidies and low interest loan for installing solar PV projects. The findings of this paper support Oman's energy sector in achieving their 2030 renewable energy goal and in framing renewable policies in the future.

## CRediT authorship contribution statement

**Sharmila deve Venkatachalam:** Writing – review & editing, Writing – original draft, Supervision, Conceptualization. **Almuhannad Al Nadabi:** Project administration, Funding acquisition. **Abdul Aziz Al Shukaili:** Software, Formal analysis, Data curation. **Ahmed Said Al Hinai:** Methodology, Investigation. **Ahmed Salim Al Shuaili:** Resources. **Ibrahim said Al Shukaili:** Visualization, Validation.

## Informed consent statement

Not applicable.

## Institutional review board statement

Not applicable.

## Data availability statement

All relevant data in the manuscript are cited in the reference.

## Funding

This research was funded by the 10.13039/100020552Ministry of Higher Education, Research and Innovation (MOHERI), grant number MOHERI/BFP/UTAS/2022.

## Declaration of competing interest

The authors declare the following financial interests/personal relationships whih may be considered as potential competing interests: Sharmila deve venkatachalam reports financial support was provided by Oman 10.13039/501100002385Ministry of Higher Education Research and Innovation. If there are other authors, they declare that they have no known competing financial interests or personal relationships that could have appeared to influence the work reported in this paper.
